# The Role of the Molecular Clock in Skeletal Muscle and What It Is Teaching Us About Muscle-Bone Crosstalk

**DOI:** 10.1007/s11914-017-0363-2

**Published:** 2017-04-18

**Authors:** Lance A. Riley, Karyn A. Esser

**Affiliations:** 1 Myology Institute, University of Florida, 1345 Center Dr., M552, Gainesville, FL USA; 2 Department of Physiology and Functional Genomics, College of Medicine, University of Florida, 1345 Center Dr., M552, Gainesville, FL USA

**Keywords:** Circadian rhythms, Muscle-bone crosstalk, BMAL1, Molecular clock, Myokines

## Abstract

**Purpose of Review:**

This review summarizes what has been learned about the interaction between skeletal muscle and bone from mouse models in which BMAL1, a core molecular clock protein has been deleted. Additionally, we highlight several genes which change following loss of BMAL1. The protein products from these genes are secreted from muscle and have a known effect on bone homeostasis.

**Recent Findings:**

Circadian rhythms have been implicated in regulating systems homeostasis through a series of transcriptional-translational feedback loops termed the molecular clock. Recently, skeletal muscle-specific disruption of the molecular clock has been shown to disrupt skeletal muscle metabolism. Additionally, loss of circadian rhythms only in adult muscle has an effect on other tissue systems including bone.

**Summary:**

Our finding that the expression of a subset of skeletal muscle-secreted proteins changes following BMAL1 knockout combined with the current knowledge of muscle-bone crosstalk suggests that skeletal muscle circadian rhythms are important for maintenance of musculoskeletal homeostasis. Future research on this topic may be important for understanding the role of the skeletal muscle molecular clock in a number of diseases such as sarcopenia and osteoporosis.

## Introduction

Physiology and behavior are temporally coordinated into rhythms coinciding with the 24-h solar cycle [1, 2]. These rhythms, termed circadian (Latin, meaning “about a day”), are present in almost every organism ranging from single cell bacteria to plants and animals [3, 4]. Underlying these rhythms in mammals is a mechanism, termed the molecular clock, which is comprised of a series of interconnected transcriptional-translational feedback loops [5]. This system functions to optimize the timing of cellular events in anticipation of environmental changes such as daylight, food availability, and predator/prey interactions. The molecular clock mechanism is found in virtually all cells within the body including both skeletal muscle and bone [6–9]. While the intrinsic mechanism is the same across cell types, the molecular clock output is highly tissue-specific [10]. Thus, the ability to synchronize the molecular clock and physiology within a tissue with external day-night/active-inactive cycles provides an evolutionarily conserved method for adapting cells to changing environmental conditions.

The classic relationship between clocks across different tissues historically focused on the top-down ability of the suprachiasmatic nucleus (SCN; termed the master clock) of the hypothalamus to govern the synchronicity of other, peripheral clock systems with its own rhythm [11, 12]. With the rise of models that uncouple the rhythm of the SCN from peripheral clock, it has become apparent that clocks in peripheral tissues are more autonomous than this traditional view [13–16]. While we know that crosstalk occurs between different tissues, the mechanisms by which clocks in one tissue might influence the physiology of another tissue has not been well studied. In this review, we present the latest findings demonstrating circadian rhythms in skeletal muscle are important for maintenance of its own cellular physiology and its effect on the physiology of other tissues, particularly focusing on the effects of disruption of the skeletal muscle molecular clock on bone health. The existing data, although limited, suggests that skeletal muscle rhythms are important for maintenance of bone [17•]. To date, there is little published work with models of bone cell clock disruption, so understanding bone to muscle crosstalk is unclear [18, 19]. These observations provide a novel role of maintenance of skeletal muscle health in the prevention of bone disease such as osteoporosis.

## The Mammalian Molecular Clock

The mechanism underlying circadian rhythms is termed the molecular clock and has been described in detail elsewhere [5, 8]. Operating as the positive arm of the core loop are two members of the PAS-bHLH family of transcription factors, CLOCK (circadian locomotor output control kaput) and *Bmal1* (brain muscle arnt-like 1) [20, 21]. These transcription factors heterodimerize and transactivate the negative limb core clock genes (*Per1/2/3* and *Cry1/2*) by binding to E-box elements (5′–CANNTG–3′) in the regulatory regions of these genes. The PER and CRY proteins then accumulate in the cytoplasm, form multimers, and translocate to the nucleus where they inhibit BMAL1:CLOCK transcriptional activity in a process that takes approximately 24 h for a complete feedback cycle.

The orphan nuclear receptors RORα and REV-ERB α/β comprise additional components of the molecular clock. These proteins affect molecular clock function by activating (RORα) or repressing (REV-ERB α/β) *Bmal1* transcription [22, 23]. Studies have also implicated kinases (e.g., CK1ε) and E3 ligases (e.g., FBXL3) associated with the proteasome system to tightly regulate the stability and accumulation of PER and CRY proteins [24, 25]. Thus, proper timing of the molecular clock mechanism requires regulation at multiple levels (i.e., transcription, translation, and posttranslational modifications) presenting many targets where environmental cues and physiological function can influence the timing of the molecular clock and cellular circadian rhythms.

In addition to their role in the molecular clock mechanism, components of the molecular clock (*Bmal1*, *Clock*, *Rev-erb*, *Rora*) have been shown to transcriptionally regulate other genes that are not directly involved in timekeeping. These downstream targets are then designated as clock-controlled genes (CCGs) [1, 26, 27]. While a subset of CCGs is similar in all tissues (e.g., *Dbp*, *Tef*), a large percentage of CCGs are tissue-specific [10]. Several tissue-specific transcription factors have been described as targets of the molecular clock [28–31]. This level of control would set up a transcriptional hierarchy within each tissue by which the core clock controls the expression of tissue-specific transcription factors which in turn acts on tissue-specific transcriptional activity [32]. In this review, we focus on what is known about the tissue-specific functions of the molecular clock in skeletal muscle and how disruption of this mechanism in skeletal muscle affects other tissues.

### The Molecular Clock in Skeletal Muscle

Studies seeking to understand the role of the molecular clock in peripheral tissues have largely involved the use of genetic mouse models targeting loss of *Bmal1*, as this is the only non-redundant gene within the core feedback loop; thus, one of the first mouse models used to study the effects of circadian rhythm disruption was the germline *Bmal1* knockout mouse (*Bmal1* KO) [21]. *Bmal1* KO mice have a shortened lifespan, exhibit features of advanced aging, and develop significant changes to bone architecture as well a variety of other pathologies [33–36]. These mice also exhibit significant skeletal muscle weakness and structural pathology in the skeletal muscle with altered myofilament architecture and abnormal mitochondrial volume and function [28]. McDearmon et al. rescued *Bmal1* in a skeletal muscle-specific manner on the *Bmal1* KO background and found that muscle *Bmal1* is sufficient to rescue many of the systemic effects found in *Bmal1* KO mice but not rhythmic circadian behavior [37]. These results argue that maintenance of the molecular clock specifically within skeletal muscle is sufficient to prevent a number of different systemic phenotypes.

In order to better understand the output of the skeletal muscle molecular clock, our lab and others have described the skeletal muscle circadian transcriptome [26, 38, 39, 40•]. Miller et al. first identified the skeletal muscle transcriptome using both WT and *Clock* mutant mice [26]. The Clock mutant mice are characterized by a long period length under constant conditions [41]. Molecular and biochemical studies determined that this mutation in *Clock* resulted in a dominant negative protein isoform and not a null mutation [42]. Comparing the total gene expression changes of these mice, this group found that both rhythmic and nonrhythmic genes were profoundly affected in the tissues of the *Clock* mutant mice. This work was followed by several publications using skeletal muscle from C57Bl/6 J and inducible, skeletal muscle-specific *Bmal1* knockout mice that identified circadian mRNAs [38, 39, 40•, 43]. These studies have confirmed the tissue specificity of the circadian transcriptome in skeletal muscle through their inclusion of known muscle-specific genes that are largely involved in metabolism, transcriptional regulation, and cellular signaling processes. More than 800 skeletal muscle enriched genes including *Myod1*, *Ucp3*, and *Myh1* display circadian oscillations in their mRNA levels suggesting a link between the molecular clock and muscle homeostasis. Since MYOD1 is both a myogenic regulatory factor and regulated by the molecular clock, it is an ideal candidate to put forth as a transcription factor to direct the tissue specificity of the molecular clock in skeletal muscle and the need for these rhythms to maintain healthy muscle.

To date, four inducible, skeletal muscle-specific *Bmal1* knockout mice have been generated to study the role of the endogenous molecular clock in skeletal muscle [40•, 43–45]. While these models use the floxed *Bmal1* mouse, they differ through the use of different muscle-specific Cre-recombinase mice. However, it is clear through the study of each of these models that skeletal muscle BMAL1 is necessary for maintenance of skeletal muscle metabolism, particularly glucose handling pathways [43, 46]. Skeletal muscle is a predominant contributor to whole-body glucose handling, and muscle insulin resistance is an early sign of metabolic syndrome. Dyar et al. were the first to report that the muscle clock regulates glucose uptake and metabolism [43]. This was followed with a study from our lab comparing gene expression between C57Bl6/J mice over time of day with an inducible, skeletal muscle-specific *Bmal1* knockout mice that found a temporal separation of carbohydrate metabolic pathways from lipid metabolic processes [40•, 46]. Most recently, Peek et al. showed anaerobic glycolysis to be regulated by the interaction of the BMAL1:CLOCK heterodimer with HIFα [44]. While each of these models have shown that the molecular clock regulates metabolic pathways in skeletal muscle, Schroder et al.’s study is the only study to date that reports the effect of skeletal muscle-specific loss of *Bmal1* on other tissues [17•].

## Skeletal Muscle-Bone Coupling

The mechanical relationship between skeletal muscle and bone has been simplified to muscle contractions serving to load and bones acting as attachment sites. This physical coupling of the two tissues is most fully appreciated in development, as they share a common mesenchymal precursor and synchronously develop based on perceived mechanical stimuli [47]. This process continues throughout the adulthood and aging, as bones change their shape and mass due to differing loads from muscle contractions and weight bearing [48]. The relationship between muscle and bone has been outlined in a mechanostat model through which osteocytes monitor deformations, partially resulting from mechanical forces from skeletal muscles, and signal osteoblasts and osteoclasts to change the architecture and mass of the bone accordingly [49]. These adaptations help to maintain homeostasis, as the bone architecture is maintained in an acceptable range. The mechanical coupling would also imply that pathologies in which muscle atrophy occurs would also result in loss of bone mass. This pairing of conditions in muscle and bone is most frequently seen in the aging population in which osteoporosis and sarcopenia are major clinical problems [50].

While the physical coupling of muscle and bone is important for bone health, there is a growing recognition of the secretory capacity of skeletal muscle. Skeletal muscle was shown to release significant amounts of interleukin-6 (IL-6) into the circulation during prolonged exercise [51]. The finding that muscle can secrete factors now termed myokines provided the conceptual framework for how muscles communicate with other organs in vivo. Since this initial study, others have tried to define the skeletal muscle secretome using a number of different models [52, 53, 54, 55, 56, 57•]. While some results differ between these models, a number of the common myokines between these studies are known to affect bone formation and resorption, including IL-6, IL-15, irisin/FNDC5, myostatin, and IGF1. The identification of myokines that affect bone growth provide the best evidence to date that the relationship between skeletal muscle and bone is both mechanical and paracrine in nature.

## The Effect of BMAL1 Knockout in Muscle on Bone

Since physical activity follows a circadian pattern, it can be assumed that at least a portion of the load on bone also follows a circadian pattern. Bunger et al. found that the germline deletion of *Bmal1* leads to progressive arthropathy but is not essential for bone development [34]. These mice also present with a decreased bone mineral density and decreased muscle force generation [28]. It can be assumed that loss of behavioral rhythmicity and thus rhythmic loading from the muscle on bone plays some role in generating this phenotype. While these findings are important to understand the relationship between these tissues, they do not allow for the study of a particular tissues contribution to the effects of the loss of circadian rhythms on bone. To date, there are limited reports of the impact of loss of *Bmal1* targeted to bone specific cell types, but the data suggests that bone resorption may be affected through modulation of RANKL expression in osteoblasts following loss of *Bmal1* [18].

Our lab’s finding that skeletal muscle-specific loss of *Bmal1* is sufficient to lead to increased bone calcification while maintaining normal cage activity highlights the importance of the skeletal muscle molecular clock in maintaining healthy bone [17•]. iMS*Bmal1* ^−/−^ mice display muscle weakness similar to germline *Bmal1* knockout mice. These mice also develop increased calcification of the Achilles’ tendon and reduced cartilage in the foot/ankle and flattened tarsals. This finding was not limited to the hindlimbs, as calcification was also observed in both the ribcage and spine. While the arthropathy is similar to what is found in germline *Bmal1* KO mice, the increase in bone calcification was unusual, since most models of muscle weakness display decreased bone calcification. These findings highlight the importance of the skeletal muscle molecular clock for the paracrine relationship between these tissues.

By analyzing published microarray data from Hodge et al., we have identified several myokines that significantly changed expression following skeletal muscle-specific knockout of *Bmal1* [40•]. Each of the secreted proteins identified in Table 1 have a known effect on bone, and the mRNA expression levels are significantly changed (*p* < 0.05) in iMS*Bmal1* ^−/−^ skeletal muscle. Below, we have highlighted the known effects of a few of these myokines and how the changed expression could result in the bone phenotype of adult mice lacking skeletal muscle BMAL1.

**Table 1:** Gene expression of several myokines with a known effect on bone formation and/or resorption change following inducible knockout of *Bmal1* in adult skeletal muscle. Data from Hodge et al. (2015) were used to identify putative myokines that could be both **a** regulated by the molecular clock and **b** have an effect on bone architecture [40•]. The data for this table came from the average of microarray data over six timepoints. Student’s *t* tests (*n* = 6/group) were then used to identify differentially expressed genes (*p* < 0.05). CircaDB (circadb.hogeneschlab.org) was used to determine if the gene was circadian in skeletal muscle using the JTK_CYCLE algorithm (*p* < 0.05) [39]

Gene symbol	Circadian in muscle (p < 0.05)	Changed in iMS*Bmal1* ^−/−^ (p < 0.05)	Bone formation	Bone resorption
Upregulated				
Fndc5	Yes	0.0457	Promotes osteoblast differentiation [58•]	
Vegfa	Yes	0.0269		Enhances osteoclast differentiation and survival of mature osteoclasts [59]
Sparc	No	0.0202	Promotes bone mineralization [60]	
Tgfb1	No	0.0142	Induce endochondral bone formation and mineralization [61]	
Ccl9	No	0.0435		Induces osteoclast accumulation at resorptive sites [62]
Anxa5	Yes	0.0124	Promotes pre-osteoblast proliferation [63]	
Ltbp4	No	0.0010	Modifies availability of TGFB1 [64]	
Thbs1	Yes	0.0232		Promotes resorption but mechanism is still unclear [65]
Igfbp4	Yes	0.0185	Inhibits osteoblast function by sequestering IGF, thus, inhibiting bone formation [66]	
Downregulated				
Il15	No	0.0178		Stimulates pre-osteoclast differentiation and decreases bone resorption by inducing natural killer cell-mediated osteoclast apoptosis [67, 68]
Mstn	No	0.0041	Negatively regulates bone formation [69]	Upregulates RANKL-induced osteoclast development [70•]
Hmgb1	No	0.0238		Potent resorption signal [71]
Igfbp5	No	0.0084	Stimulates bone formation and may function independently of IGFs [72]	Stimulates osteoclast function by augmenting IGF-1 [72]
Gdf11	Yes	0.0010	Inhibits osteoblast differentiation [73]	Stimulates osteoclastogenesis [73]

### Myostatin

Myostatin is a muscle-specific member of the transforming growth factor β superfamily of proteins that is secreted from muscle and is most known for negatively regulating muscle size [74]. Recently, inhibition of the myostatin pathway was shown to increase bone turnover and increased bone mass [69]. Inhibition of the myostatin receptor (ActRIIB) in osteoblasts leads to increased bone formation in mice [75]. Myostatin is also linked to RANKL-induced osteoclast development through promoting the expression of the transcription factor NFATC1 [70•]. Inhibition or loss of myostatin strongly reduces osteoclast formation and bone destruction. Thus, the effect of myostatin on bone is two-fold. It first acts to inhibit osteoblast function through ActRIIB then activates bone resorption through its promotion of osteoclast formation. iMS*Bmal1* ^−/−^ mice display a 30% decrease in myostatin mRNA expression which could explain in part the increased calcification seen in these mice.

### IGFBP5

IGFBP5’s role in bone formation is largely known through its ability to bind to the extracellular matrix allowing for IGFs to bind to the surface of bone cells. It is the only IGFBP that has been consistently shown to stimulate osteoblast proliferation [72, 76]. IGFBP5 has also been reported to stimulate osteoclast bone resorption in the presence of osteoblasts [77]. While many of the effects of IGFBP5 on bone formation are positive, overexpression of this protein using an osteocalcin promoter led to decreased bone volume [78]. Thus, it is likely that IGFBP5 works in a dose-dependent manner to regulate bone formation.

There is a 29% decrease in IGFBP5 gene expression following skeletal muscle-specific knockout of *Bmal1*. The phenotype of these mice could be caused by this change due to a change in the dose of IGFBP5 available on the bone surface. Perhaps at lower doses, IGFBP5 stimulates osteoblast proliferation leading to an increased bone density, while at higher doses, it would lead to an increased stimulation of resorption. Additionally, this protein is positively correlated with growth and bone formation such that it increases during development but decreases in aging and osteoporosis. The apparent decrease in IGFBP5 following skeletal muscle loss of *Bmal1* is thus consistent with the idea that the iMS*Bmal1* ^−/−^ mouse is a model of advanced aging.

### TGFB1

Transforming growth factor beta 1 is known to be important for the maintenance and expansion of mesenchymal progenitor cells and osteoblasts through the selective MAPKs and SMAD2/3 pathways [61]. TGF-β1 has been shown to promote matrix production and osteoblast differentiation. In fact, TGF-β1 deficient mice present with reduced bone growth and mineralization [79]. Additionally, this protein reduces osteoblast’s ability to secrete RANKL, a potent osteoclast differentiation factor, limiting osteoclast formation and thus affecting bone mass [80]. TGF-β1 is also a common signaling molecule in a variety of other pathways such as the Wnt signaling pathway in osteoblasts and bone morphogenic protein (BMP) signaling pathway in osteocytes [61]. Thus, the effects of increased amounts of this protein in the circulation should lead to increased bone formation and decreased bone resorption.

A chronic increase in TGFB1 expression by muscle could act in a paracrine fashion to contribute to the bone phenotype seen in iMS*Bmal1* ^−/−^ mice. Since this protein is known to promote bone formation and limit osteoclast formation, it might play a role in the increased calcification of bone in this model. Additionally, the convergence of the TGF-β1 signaling pathway with BMP signaling pathways could explain the reduced cartilage staining and increased calcification of the Achilles’ tendon, as this pathway is known to lead to heterotopic ossification.

### Irisin

Irisin is a recently discovered myokine that is cleaved from fibronectin type III domain-containing protein 5 (FNDC5), a membrane-bound protein in skeletal muscle that is induced by exercise [81]. This myokine has been primarily studied through its autocrine functions regulating muscle metabolism and endocrine functions leading to the beiging of white adipose tissue; however, it has recently been shown that it also plays a role in regulating osteoblast function [82, 83]. Colaianni et al. showed that at low doses, this hormone-like myokine enhances osteoblast differentiation and increases cortical bone mineral density; thus, it positively modifies bone geometry [58•].

*Fndc5*, the precursor gene for irisin, increases nearly 14% in iMS*Bmal1* ^−/−^ mice. While this change is small, we believe that a chronic increase in FNDC5 would lead to biologically significant changes on the bone architecture. Since irisin is known to enhance osteoblast differentiation and modify bone geometry, its increase in iMS*Bmal1* ^−/−^ mice could contribute to both the observed increase in calcification as well as the flattening of the tarsals and misshaping of the tibia and fibula of iMS*Bmal1* ^−/−^ mice.

### GDF11

Growth differentiation factor 11 (GDF11) is a member of the TGF-β superfamily of proteins whose deletion results in abnormal skeletal patterning during development [84]. There has been significant controversy over the past 3 years regarding GDF11 and its role in muscle aging and regeneration. Initial reports suggested circulating GDF11 levels decline with age-making strategies that increase GDF11 potentially therapeutic [85–87]. This concept, however, has been challenged by the findings of several different labs [88–90]. Most recently, it was shown that overexpression of GDF11 in mice induces muscle atrophy, inhibits skeletal muscle regeneration, leads to bone loss, and blocks bone resorption; all outcomes that argue that increasing GDF11 would not be a beneficial strategy for musculoskeletal health [73, 89, 91, 92]. We found that the average expression of *Gdf11* decreases approximately 17% in iMS*Bmal1* ^−/−^ mice 5 weeks post knockout of *Bmal1*. We did not measure circulating GDF11 levels, and while the decline in *Gdf11* mRNA expression in the iMS*Bmal1* ^−/−^ muscle might mimic changes with age, it is unclear whether these changes are contributing to the bone phenotype seen in these mice.

## Conclusions

The role of the endogenous molecular clock in peripheral tissues, such as skeletal muscle, is a rapidly emerging area of research. Recent studies using different models to induce *Bmal1* knockout specifically in adult skeletal muscle have concluded that maintenance of circadian rhythms in skeletal muscle is necessary for metabolic homeostasis in skeletal muscle [40•, 43–45]. Since these models have normal behavioral (i.e., activity) rhythms, skeletal muscle-specific *Bmal1* knockout mice provide a good model for looking at changes in signaling between muscle and bone without affecting rhythmic loading. Schroder et al. reported changes in the bone calcification and joint cartilage deposition in iMS*Bmal1* ^−/−^ mice that is consistent with the increased bone calcification seen in the germline *Bmal1* KO mice [17•, 34]. While muscle weakness is usually accompanied by decreases in bone calcification, this model’s altered bone architecture suggests the changes are more likely due to disrupted cytokine/myokine circulation. Using microarray data from this model, we have identified 14 myokines with a known effect on bone homeostasis whose gene expression in skeletal muscle significantly changes following *Bmal1* deletion [40•]. These findings suggest that skeletal muscle BMAL1 is important for maintenance of bone health and highlight the importance of skeletal muscle circadian rhythms in musculoskeletal homeostasis, with implications for aging [93]. Increased knowledge of the relationship between the skeletal muscle molecular clock and muscle-bone crosstalk could lead to a better understanding of aging-related diseases such as sarcopenia and osteoporosis. Additionally, uncovering the pathways underlying this relationship could lead to time of day-based intervention strategies (e.g., exercise and dietary restriction), which alter clock genes in muscle to promote healthy aging.
